# Developing the specifications of an Open Angle Glaucoma screening intervention in the United Kingdom: a Delphi approach

**DOI:** 10.1186/1472-6963-12-447

**Published:** 2012-12-05

**Authors:** Susan E Campbell, Augusto Azuara-Blanco, Marion K Campbell, Jillian J Francis, Alexandra C Greene, Craig R Ramsay, Jennifer M Burr

**Affiliations:** 1 School of Nursing Sciences, Edith Cavell Building, University of East Anglia, Norwich Research Park, Norwich, UK; 2 Health Services Research Unit, University of Aberdeen, Health Sciences Building, Foresterhill, Aberdeen, UK; 3 Child Health, University of Dundee, MACHS Building, Tayside Children’s Hospital, Ninewells Hospital and Medical School, Dundee, UK

## Abstract

**Background:**

Glaucoma is a leading cause of blindness. Early detection is advocated but there is insufficient evidence from randomized controlled trials (RCTs) to inform health policy on population screening. Primarily, there is no agreed screening intervention. For a screening programme, agreement is required on the screening tests to be used, either individually or in combination, the person to deliver the test and the location where testing should take place. This study aimed to use ophthalmologists (who were experienced glaucoma subspecialists), optometrists, ophthalmic nurses and patients to develop a reduced set of potential screening tests and testing arrangements that could then be explored in depth in a further study of their feasibility for evaluation in a glaucoma screening RCT.

**Methods:**

A two-round Delphi survey involving 38 participants was conducted. Materials were developed from a prior evidence synthesis. For round one, after some initial priming questions in four domains, specialists were asked to nominate three screening interventions, the intervention being a combination of the four domains; target population, (age and higher risk groups), site, screening test and test operator (provider). More than 250 screening interventions were identified. For round two, responses were condensed into 72 interventions and each was rated by participants on a 0-10 scale in terms of feasibility.

**Results:**

Using a cut-off of a median rating of feasibility of ≥5.5 as evidence of agreement of intervention feasibility, six interventions were identified from round 2. These were initiating screening at age 50, with a combination of two or three screening tests (varying combinations of tonometry/measures of visual function/optic nerve damage) organized in a community setting with an ophthalmic trained technical assistant delivering the tests. An alternative intervention was a ‘glaucoma risk score’ ascertained by questionnaire. The advisory panel recommended that further exploration of the feasibility of screening higher risk populations and detailed specification of the screening tests was required.

**Conclusions:**

With systematic use of expert opinions, a shortlist of potential screening interventions was identified. Views of users, service providers and cost-effectiveness modeling are now required to identify a feasible intervention to evaluate in a future glaucoma screening trial.

## Background

Glaucoma is a chronic progressive eye disease and is the leading cause of irreversible blindness worldwide [1]. Open Angle Glaucoma (OAG) is the most common form of glaucoma [2]. OAG is asymptomatic until advanced stages of the disease but if identified early, treatment can be relatively effective at reducing the rate of progression [3]. In the UK, glaucoma is detected by opportunistic case finding usually bay an optometrist. Tests for glaucoma involve assessment of structural changes at the optic nerve head by fundoscopy or imaging, functional visual loss by visual field testing and the level of intraocular pressure. Of an estimated half a million people affected in the UK, estimates from population based studies suggests that less than half have been diagnosed [4]. A population screening programme for OAG has been proposed as a policy to reduce the burden of glaucoma; however, before a screening programme is introduced robust evidence from high quality randomized controlled trials (RCTs) is required to demonstrate that any benefits of screening on reducing sight loss outweigh any potential harms and that this is cost effective [5].

The available literature on the clinical and cost-effectiveness of screening for OAG was summarized in a recent health technology assessment (HTA) [6,7]. No RCTs of screening were identified [6]. Prior to the conduct of any large definitive screening trial, evidence is required to develop the components of the screening interventions to be tested. The HTA report identified uncertainties regarding the clinical components of the intervention namely: the selection of optimal screening approaches (including the choice of screening tests); how and where screening should be provided and which healthcare professionals would administer the test; whether the screening process should be limited to individuals in the ‘at risk’ groups, (e.g. those with a family history of glaucoma in a first degree relative or those of black ethnicity) or to screening based on age criteria alone.

Screening tests should be relatively simple, safe and acceptable to the population being tested and sufficiently sensitive and specific to distinguish those who do or do not have the condition. Facilities should be in place for those screening positive to have further testing to establish a diagnosis [5].

The process of intervention development requires that the intervention is feasible for both evaluation in the RCT and importantly for any future implementation in a policy context. Thus, in addition to likely future effectiveness identified from evidence synthesis, selection of the optimal glaucoma screening intervention rests on the feasibility and in a service context and on economic considerations.

These criteria may be explored using a range of research methods. The Delphi method provides opportunity for experts to communicate their opinions and knowledge about a complex problem in order to explore options and potentially reach consensus even if they are in geographically dispersed areas. It has application in many fields such as healthcare, education and sociology [8]. It can also help to remove the bias of face to face consensus meetings where interaction can sometimes lead to those with a stronger opinions dominating the decision making process [9-11].

The process is repeated until a level of agreement is reached [9]. This is achieved through a series of questionnaires (known as ‘rounds’) together with feedback about questionnaire data from earlier rounds [12]. There are no strict guidelines on the most appropriate number of rounds as this is a function of the complexity of the issues to be decided and the heterogeneity of the sample recruited. However, it generally ranges from two to four rounds [11-14].

We report a two round Delphi process with experts in the area of glaucoma screening. The purpose of the Delphi was to reduce the many potential components of a screening intervention, in terms of who to screen and how in terms of optimal testing schedule, to a reduced set of potential interventions that could be explored in depth in a qualitative enquiry within a UK National Health Service (NHS) context. The qualitative enquiry has been reported elsewhere [15].

## Methods

Taking evidence from a prior evidence synthesis [8] a two-round Delphi process was conducted. Results were then reported to the project advisory panel for discussion and critique of the results. The results including suggestions from the project advisory panel were taken forward to the next stage of the research [15,16].

### Group selection

We sampled purposively to include a range of relevant health professional groups (ophthalmologists, optometrists and ophthalmic nurses) as well as patient representation in the UK and internationally [9]. Sampling was weighted in favor of ophthalmologists from the UK (due to their expert knowledge of the NHS system where the proposed trial and future screening policy would be implemented). Ophthalmologists were selected who were subspecialists in glaucoma and had published in the field (in order to examine the views of those treating glaucoma, about the best screening strategies). Those ophthalmologists selected as International experts were members of the World Glaucoma Association consensus panel for screening for glaucoma. Optometrists were selected as they would have additional views particularly on the administration of the tests, nurses who had training in ophthalmology and patients who were representatives of the International Glaucoma Association.

### Measures and procedures

The first-round questionnaire was sent with a participant information sheet and an explanation of the attributes of potential glaucoma screening tests to 38 potential participants. The screening tests chosen, and the detail on each test, were based on evidence from a systematic review, and from subsequent reports on population based screening studies [4,6,7,17-24]. There were three tests of visual function, three tests of optic nerve damage and three alternative methods of measuring intraocular pressure (tonometry). (For details of the screening test attributes see Additional file 1).

The questionnaire consisted of four sections. First, participants were invited to rate tests of visual function and structural loss and intraocular pressure on a scale between 0-4 with 4 indicating that the test was ‘very suitable’ as part of an intervention to be evaluated in a future RCT and 0 indicating that it was ‘not suitable’. Second, to suggest one or more tests to be used to screen for glaucoma; third to give their views on the target population, such as age, risk factor, whether screening should be based on age alone and whether screening tests should vary according to the population being screened; and fourth, to suggest the screening site and operator, e.g. the home, mobile van or General Practitioner and who should provide the test, e.g. a nurse, ophthalmologist, optometrist, ophthalmic trained technical assistant, as well as the combinations of where the screening should take place and by whom. These four parts to the questionnaire were essentially to help focus participants into, finally, listing their top three combinations of screening interventions within each of the four domains:, tests, population, provider and site, to be evaluated in the proposed RCT. Free text boxes were provided to allow participants to add additional comments.

Results from the first round questionnaire for each section of testing strategy, target population, screening site and operator were fed back to 31 (82%) of the 38 participants who completed round one and two participants who were unable to complete round one but could take part in round two (Additional file 2, feedback to participants). Then the second questionnaire presented the combinations of screening interventions which each participant had identified in the final part of the round one questionnaire. As there were more than 250 combinations we condensed responses into common themes. For example for the location of the test, mobile van, the General Practitioners’ surgery, or a retail setting were represented as a community-based “visual screening centre” (VSC). This condensing process resulted in a questionnaire consisting of 72 combinations being sent to participants. Eight of these combinations were chosen by more than one participant and were automatically included in the second round (Table 1). An asterisk next to the eight combinations on the second questionnaire indicated that it had been chosen by more than one respondent in round one.

**Table 1:** Combinations from round one suggested by more than one respondent

**Population**	**Site**	**Provider**	**Test**
60 years	vision screening centre	technician*	SAP
50 years	hospital	nurse	GAT and fundoscopy
50 years	vision screening centre	technician*	tonometry (Icare or tonopen or NCT) and FDT
50 years	optometry practice	optometrist	GAT and fundoscopy
50 years	optometry practice	optometrist	GAT and FDT
40 years for those with FH or of black ethnicity	vision screening centre	technician*	SAP (non specified) repeat test required
40 years for those with FH or of black ethnicity	vision screening centre	optometrist or technician*	GAT and angle assessment, HRT and visual function test
40 years for those with FH or of black	vision screening centre	technician*	FDT

* ophthalmic trained technical assistant *FDT* – Frequency doubling perimetry *GAT* - Goldmann applanation tonometry *HRT*-Heidelberg Retina Tomograph.

*NCT* – Non-contact tonometry.

*SAP* – Standard automated perimetry.

*FH* - First degree relative with glaucoma.

**Figure 1:**
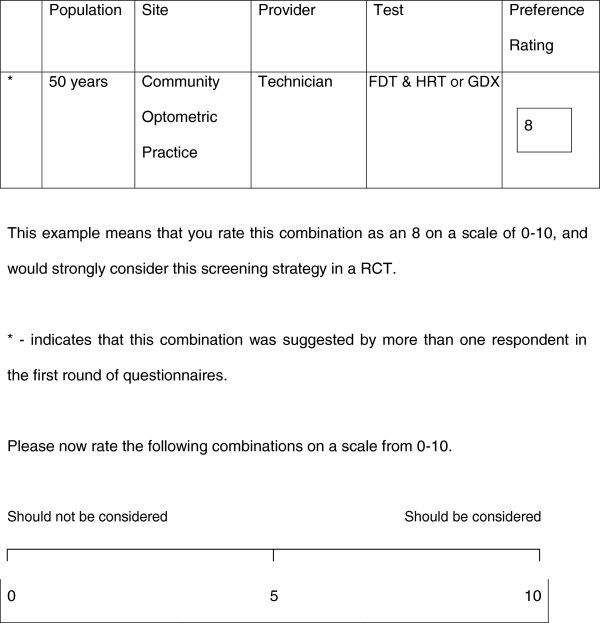
**Example combination and instructions on how participants were asked to complete the questionnaire (Round 2).** This example means that you rate this combination as an 8 on a scale of 0-10, and would strongly consider this screening strategy in a RCT. * - indicates that this combination was suggested by more than one respondent in the first round of questionnaires. Please now rate the following combinations on a scale from 0-10.

Figure 1 displays an example test combination and instructions that were given to participants for completing the questionnaire. The round two questionnaires were sent to those who agreed to participate in this next round, with the detailed report of round one, as well as an explanation of the attributes of tests of visual function, the same as the round one material (Additional file 1). The 72 testing combinations described in the feedback portion of questionnaire specified the target population, the site where the screening could take place, who would conduct the screening and finally the tests which would be carried out. Participants were asked to rate their preferences on a scale of 0 (the test should not be considered) to 10 (should be considered) as the screening intervention to be evaluated.

### Data analysis

The first round percentage agreement is presented, below, for each section of the questionnaire. For the second round it was agreed a priori to include all combinations with a median above 5 (on the 0 – 10 point scale). Therefore for each of the 72 combinations, the median of the participants’ scores was calculated and the combinations with median scores of 5.5 and over were presented and discussed with the advisory panel before being taken forward to the next phase of research [15]. The advisory panel of 12 people included ophthalmologists, optometrists, an ophthalmic nurse, a patient and a patient organization representative.

This study was classified as a service evaluation and did not require national research ethics committee approval (as advised by the North of Scotland Ethics Committee). We certify that all applicable institutional and governmental regulations concerning the ethical use of human volunteers were followed during this research.

## Results

### Round 1

Thirty-one of 38 (82%) questionnaires were returned in round one. However, two who were unable to be involved in the first round had indicated their willingness to be involved in round 2. Therefore, the round 2 sample consisted of 33 participants and 23 (70%) questionnaires were returned. Respondents consisted of 15 ophthalmologists, 6 optometrists, 1 ophthalmic nurse and 1 service user. The majority of respondents were from the UK due to the sampling strategy but Europe, USA, Canada and Australia were also represented.

The main results for round 1 are summarized in the text for more detailed tables these are detailed in Additional file 2 Feedback to participants from the round one Delphi.

Twenty nine (93%) indicated that a test of visual function should be considered, 28 (90%) a test of structural loss and 28 (90%) a measure of intraocular pressure. However, there was wide variation in rating of which test in each of these categories was most suitable for use. (See Additional file 2).

For the age when screening should start, one participant (3%) would screen at 30 years of age for those of black ethnicity. Nine (29%) would begin screening for those in their 40s but the majority, 17 (55%) recommended 50s with four (16%) recommending 60 years of age.

Fourteen (45%) would screen on age alone as opposed to age and an additional risk factor, 12 (39%) would screen on age alone with an additional risk factor and five (16%) were unsure. Respondents who recommended screening on age and another risk factor, 24 (77%) identified having a parent with OAG, 23 (74%) a sibling with OAG, 22 (71%) people of black ethnic group, as the important risk factors. When asked if the screening tests would vary according to the population screened only 8 (26%) respondents would vary their choice of tests according to the population being screened.

The majority indicated suitable (rated 3 and 4 on the 0-4 scale) sites to administer the tests were community optometric practice (24, 78%), mobile van (14, 46%) or general practice (13, 42%). Several novel sites were also suggested, open access care centers for those not registered with a General Practitioner, shopping malls, church or community centers. The top test operator (rated 4, very suitable) was technician (15, 48%), community optometrist (12, 39%), specialist glaucoma trained optometrist (12, 39%), nurse (8, 26%) and only one (3%) rated self testing as very suitable.

### Round 2

**Figure 2:**
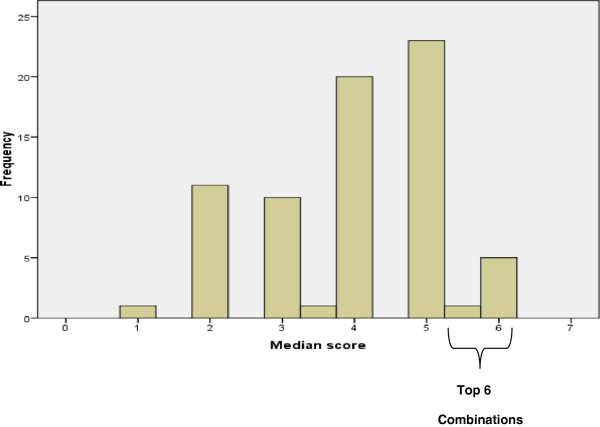
Distribution of median scores for every screening strategy combination.

Based on the reported responses the participants were then asked to rate the 72 combinations in terms of their feasibility as screening interventions for evaluation in a future screening trial. Figure 2 shows the median scores for every screening strategy combination. The top six combinations (median of 5.5 and over) are detailed in Table 2. There was evidence of consensus for initiating screening at age 50 and for screening taking place in a community setting (either the optometrists or a community visual screening centre (VSC), with ophthalmic trained technical assistants delivering the screening tests which were all rated individually as very suitable in round 1. Tailoring screening to higher risk groups was not short-listed. However, one combination did include a questionnaire to identify at-risk groups.

**Table 2:** Shortlisted interventions from round two

**Population**	**Site**	**Provider**	**Test**	**Median**
50 years	Optometry Practice	Technician*/Optometrist	NCT, SAP (multiple stimuli) and HRT	6
50 years	Optometry Practice	Technician*	Icare tonometry, FDT, and disc photography	6
50 years	VSC	Technician*	Tonometry (Icare or tonopen or NCT) and FDT	6
50 years	VSC	Technician*	GAT, SAP and disc photography	6
50 years + risk score	VSC	Technician*	Tonometry and rapid reproducible imaging or visual function test	6
50 years	VSC	Technician*	NCT and FDT and HRT or GDx	5.5

* ophthalmic trained technical assistant.

Risk score - based on self identified risk factors by questionnaire sent to the screening cohort - including family history, black ethnic group, myopia or no prior visits to eye care services.

*VSC* – Vision Screening Centre.

*FDT* – Frequency doubling perimetry *GAT* - Goldmann applanation tonometry *GDx* - Scanning laser polarimetry.

*HRT* – Heidelberg retinal tomograph.

*NCT* – Non-contact tonometry.

*SAP* – Standard automated perimetry.

The selected top combinations were a combination of the individual preferences in round one for screening starting at age 50, in a community setting, by a technician. A wide range of tests for the screening intervention were identified and round one had highlighted some uncertainties around the tests from free text comments (section “**Quotes from the round one Delphi questionnaires showing reasons for lack of consensus about tests”**) which seems to be reflected in the different tests chosen.

Choice of screening strategy

‘It is my belief that threshold field tests are inappropriate for screening

(should be reserved for diagnosis) because they are associated with considerable false positives (and probably false negatives).’

‘Simple functional test should be used’

‘Any screening programme for glaucoma must include a field test.’

‘Technicians are better at following protocols than doctors’.

‘I rated certain combinations low because I don’t believe the method has any value in any usage (i.e. Icare tonometer).’

‘I am very uncertain about tonometry in screening-do we want to detect ocular hypertension or glaucoma? If we test for ocular hypertension we will generate huge numbers of false positives for glaucoma but who do have ocular hypertension (many of whom will have a thick cornea). Though radical, I think we should exclude intraocular pressure, on balance, since it is such a poor test for glaucoma.’

‘The screening programme must be feasible in terms of cost of equipment and paying for technicians, which eliminates almost everything except Non Contact Tonometry, and computer based solutions such as Frequency Doubling Technology, Motion Detection Technology and Oculo-Kinetic Perimetry.’

### The advisory panel

These results were then presented to the advisory panel. The panel agreed that the six short-listed interventions would be combinations which would be worth taking forward to explore in our interviews with providers in the next part of the research [15,16]. It was noted that many of the chosen tests were not screening tests but diagnostic tests, in that the initial test for screening is not supposed to give the definitive answer but to identify those who are unlikely to have glaucoma and move those with a positive result for further scrutiny and diagnosis [24]. The advisory panel recommended that during the interviews a single technology based test ± tonometry as the screening intervention should also be explored, with the battery of tests identified in round two as the diagnostic strategy for people whose test result was positive on the initial screening.

The panel noted that the six combinations did not include a combination for identifying at risk groups, (those with a family history of glaucoma in a first degree relative or those of black ethnicity), apart from a screening questionnaire. It was recommended to explore how best to target the higher risk groups in the coming interviews highlighted above. The panel recommended that other vulnerable groups at greater risk of going blind from late detected glaucoma are those from low socio-economic groups, low education groups and those who are housebound and known not to have easy access to eye care services. A few members of the panel argued that the greatest risk was linked to late or no contact with eye care services and social deprivation rather than to black ethnicity. For all members the main problem proposed was identifying interventions that would encourage those at risk to attend for screening. This would be particularly difficult for at risk groups, such as Afro-Caribbean groups, where the risk of open angle glaucoma may be greater than for other African groups. Most members felt it would be more acceptable and feasible to identify the target population by age, geography and social deprivation scores for example by post code, electoral roll and General Practice records.

## Discussion

The Delphi panel of experts identified six screening intervention combinations to take forward to the next phase of the research. All included starting screening at age 50 years with one using an additional risk score to identify at risk groups. Most identified the site as a community based visual screening centre which included, general practice, mobile van or retail outlet, with the other combinations identifying community optometry. All combinations included an ophthalmic trained technician as the test provider with one combination also including an optometrist. Opinions about tests were less certain but most included a measure of intra ocular pressure.

The response rate to our Delphi survey for both rounds was high. Main studies detailing the method argue that rigor can be maintained with a response rate of 70% which we have reached [8,10]. Our panel was made up of a range of experts in the field of ophthalmology. Mainly UK based as this was related to provision within the NHS. Our panel of international experts was small and may not include the views of all with an interest in glaucoma screening.

There are no universally agreed criteria in Delphi methodology for the selection of experts and there are variations in the guidance on the minimum or maximum number of experts required [25]. What appears to be important is common sense, and practical logistics to gain coverage of relevant expertise, not based on numbers but on the quality of the group, to ensure specialist consideration of a wide range of views [14]. Using the purposive sampling approach helped to achieve agreement in most of the components and the range of tests reflects the differences in clinical opinion which does require further exploration. Having a further advisory panel which included a range of experts and a service user helped to provide another clinical view as well as explore other areas of importance (such as at risk groups) and the possibilities for further study. However, we do acknowledge the potential for bias in the experts and group selected.

Although a Delphi process removes some of the problems of interaction it also can remove some of the benefits of an exchange of information [25]. Therefore, reporting the results to the advisory panel, the further discussion helped to confirm that, given the number of tests selected, some participants appeared to be identifying a diagnostic strategy rather than a screening strategy. There was also recognition that high-risk groups had not been shortlisted. They advised that there should be further exploration of the tests and at risk groups through the qualitative interviews in the next phase of the intervention development [15,16].

There is debate about what initial percentage agreement demonstrates consensus. We selected those with a median of 5.5 and above on our 0-10 scale and this is supported in other studies where consensus or agreement can range from a starting percentage of 51% to 70% [14,26,27].

It may be argued that using an advisory panel as well as doing a Delphi survey made our study complicated and that the Delphi study was in some ways over ruled by the panel. However, we believe that both approaches enhanced our work in that we gained both a clinical perspective, evidence based perspective as well as a practical approach to the next stage of the study.

The findings from this study are consistent with the World Glaucoma Association (WGA) consensus guidelines on glaucoma screening [28]. The guidelines highlight that the best single test or group of tests for OAG screening are not yet determined and it was noted that the tests that are available and effective for case- finding are not necessarily the same as those for population screening. The WGA consensus guidelines note that although the best evidence to date suggests screening of high risk subgroups is more cost-effective it also suggests that population-based screening studies are required to determine optimal screening strategies and their cost-effectiveness [28]. The WGA guidelines process did not use formal consensus methodology and the evidence to underpin the guidance was not identified or synthesized systematically.

## Conclusions

This study has used an established method for the systematic use of expert opinion, informed by prior Health Technology Assessment reviews [4,6,7]. It informs the choice, from the many potential screening test interventions, of those interventions most likely to be feasible for population screening for glaucoma. The next step of this research is to explore the feasibility and acceptability of these interventions to both service providers and users and to evaluate their potential cost-effectiveness. This phased approach develops the methodology for complex interventions in the context of a potential glaucoma screening trial.

## Competing interests

The authors declare that they have no competing interests. 

## Authors' contributions

JMB, MKC, JF, AG, and CRR had the original ideas for the study. All authors developed the protocol. SEC, AG, JB, CRR conducted the Delphi survey and performed the analysis. AAB gave clinical advice on each round of the Delphi process. All authors contributed to revisions of the paper.

## Pre-publication history

The pre-publication history for this paper can be accessed here:

http://www.biomedcentral.com/1472-6963/12/447/prepub

## Supplementary Material

Additional files 1 **Attributes of candidate glaucoma screening tests.** An explanation of the attributes of tests of visual function to be considered as part of a glaucoma screening intervention. Click here for file

Additional file 2 **Feedback to participants from the round one Delphi.** A summary of the responses from round one sent to all participants with their round two questionnaires. Click here for file
