# Supplementary material for: Developing the specifications of an Open Angle Glaucoma screening intervention in the United Kingdom: a Delphi approach
Source: BMC Health Serv Res. 2012 Dec 5;12:447. doi: 10.1186/1472-6963-12-447 (PMC3563574; doi:10.1186/1472-6963-12-447)
Supplement: Additional files 1 — Attributes of candidate glaucoma screening tests. An explanation of the attributes of tests of visual function to be considered as part of a glaucoma screening intervention. [file 1472-6963-12-447-S1.pdf]

Attributes of candidate glaucoma screening tests*

Screening tests	*Test attributes*
**Visual Function**<br>*Frequency doubling perimetry (FDT)*	• **Duration of test**: About 8 minutes per person tested (assuming that two tests are done on each eye);<br>• **Technical detail**: With training a technician can operate the test Portable (relatively light and easy to set up), a technician can operate the test. Refractive correction is generally not required except for those with high refractive error and then their own spectacles can be worn for the test.<br>• **Validity**: Across all populations, FDT performs with a sensitivity of 92% and specificity of 94%, with 98% of those undertaking the test reported as being able to provide an interpretable result. [1] However, recent evidence using FDT in a Japanese context to screen the general population suggests that in a screening context the sensitivity and specificity are much lower at 56%, 93% respectively. As expected the sensitivity increased if one aimed to detect glaucoma with a moderate visual field defect. 96% of those tested were able to provide a readable result. [2] A separate study in a Chinese population reported that 5% of eyes (450/8615) were truly false positives in that they failed the FDT screening test and no reason (no glaucoma or other pathology) for an abnormal result was found.[3] Evidence suggests that initially abnormal screening tests should be repeated; screening using FDT in community locations in the USA found that of those with abnormal FDT tests, 30% (575/ 1918) returned to normal on repeat testing. [4]
Oculo-kinetic perimetry (OKP)	• **Duration of test:** About 16minutes per patient, (assuming that two tests are done on each eye);<br>• **Technical details**: A technician can operate the test and self testing is feasible**.**<br>• **Validity**: Across all populations, OKP performs with an estimated sensitivity of 86% and specificity of 90%, with 97% of those undertaking the test reported as able to provide an interpretable result.[1] However, this is based on limited evidence in a population screening setting.<br>• **Other**: OKP with the Damato chart is a simple and inexpensive visual field test device. A modified version was available (free of charge) on the Internet http://www.testvision.org, although this does not at present appear to be available.
Standard automated perimetry (SAP)	• **Duration of test**: About 30 minutes per patient, (assuming that two tests are done on each eye);<br>• **Technical Detail**: With training a technician can operate the test.<br>• **Validity**: Across all populations, SAP, suprathreshold and threshold, performs with an estimated sensitivity of 71-88% and specificity of 80-85%, with 98% of those undertaking test reported as able to provide an interpretable result.[1] However this is based on limited evidence in a population screening setting; elderly and infirm patients have difficulty engaging in the test, but if screening was focused on the younger age groups (most likely to benefit from screening) then this may not be a problem.<br>• **Other**: SAP does have the advantage of its availability in that most community optometric practices in the UK are equipped with automated perimeters.

**Structural loss (evaluating glaucomatous damage to the retinal nerve fibre layer and optic nerve head)**	
Digital Photography (either monoscopic or stereoscopic and including retinal nerve fibre layer photography)	• **Duration of test**: About 30minutes per patient;<br>• **Technical detail**: With training a technician can operate the test. Pupil dilation is required if stereoscopic images are required.<br>• **Validity**: Across all populations, photography performs with an estimated sensitivity of 73-75% and specificity of 80-89%, with 80-85% of those undertaking test reported as able to provide interpretable result.[1]<br>• **Other**: The technology is available particularly through the diabetic retinopathy screening programme. Interpretation of photography is subjective and relies on the examiners experience.
Heidelberg Retinal Tomography (HRT)	• **Duration of test:** About 30 minutes per patient.<br>• **Technical detail**: With training a technician can operate the test.; specific training is required in contour line placement for HRT-II, which can be a source of error in image analysis. Pupil dilation is not required, and cataracts do not prevent imaging.<br>• **Validity**: Across all populations, HRT-II performs with an estimated sensitivity of 55-97% and specificity of 66-98%, with 91-97% of those undertaking test reported as able to provide interpretable result<br>• **Other**: The HRT-III incorporates a new, larger, race-specific database. The software V.3.0 calculates the Glaucoma Probability Score (GPS), a new, automated algorithm that evaluates both optic disc and parapapillary RNFL topography to estimate the probability of glaucoma. No contour line or reference plane is used in the GPS calculation, and therefore it is completely an operator-independent analysis. For HRT II or 111 to be used in a screening situation the diagnostic criteria for classification as normal and abnormal need to be appropriately defined. It is worth noting that the performance of the *HRT and all imaging devices (applies to GDxVCC) is* dependant on the optic disc size and eyes with optic disc abnormalities, such as large myopic discs with peripapillary atrophy cannot be imaged reliably. [5,6]
Scanning Laser Polarimetry<br><br>*-the GDX VCC*	• **Duration of test**: About 20minutes per patient<br>• **Technical Detail**: With training a technician can operate the test, pupil dilation is not required.<br>• **Validity**: Subsequent to the HTA report[1] A population study in Hungary reports, in a self-recruited population with relatively high risk for mild glaucomatous damage, that GDx-VCC (Nerve Fibre Indicator threshold of >30) performed with 97.0% specificity and 25.6% sensitivity; sensitivity improved when a matrix-FDT test was added .[7]
**Measurement of Intraocular pressure (IOP)-Tonometry**	• **Validity**: Across all populations, Tonometry at a cut-off of >21mmHG, performs with an estimated sensitivity of 46% and specificity of 95%, with 99% of those undertaking test reported as able to provide interpretable result.[1]
Contact tonometry	• **Duration of test** :1-2 minutes per eye<br>• **Technical detail**: Goldmann Applanation tonometry (GAT) is the most widely used tonometer by ophthalmologists. A clinical background is not essential for doing the test; with training a technician can do GAT. GAT is mounted onto a slit-lamp which limits its convenience. The Perkins

	tonometer is a portable device that is similar to the GAT and maybe more suitable as a screening device. Tonopen is an automated hand-held applanation tonometer it is small, light, and battery operated. Contact tonometry requires the use of topical anaesthesia applied to the cornea.<br>• **Reliability**: Unusually thick or thin corneas lead to measurement error. Data were not available on the reliability of Tonopen or Perkins tonometry in a screening situation.<br>• **Other**: There are potential problems with using contact tonometry in a screening situation in that contact with the tear film and the cornea may raise concerns regarding transmissible disease. Chemical disinfection is required after each test to reduce the risk of cross infection. Disposable prisms for Goldmann and Perkins tonometry or disposable covers for the Tonopen tip avoid the risk of cross-infection.
Non-contact tonometry (NCT)	• **Duration of test** – 1-2 minutes per eye<br>• **Technical Detail**: A small hand held and portable device, intended for use by general practitioners and optometrists and does not require corneal anaesthesia. With minimal training a technician could operate the test<br>• **Reliability**: Measurement errors are larger in patients who squeeze their eyelids or who blink rapidly in response to the startling jet of air. Unusually thick or thin corneas lead to measurement error.<br>• **Other**: There is no direct contact with the eye and thus infection control issues are avoided.
Rebound tonometry- Icare tonometer	• **Duration of test** : 1-2 minutes per eye<br>• **Technical Detail**: A small hand held and portable device, intended for use by general practitioners and optometrists, use of the device does not require topical corneal anaesthesia and it is potentially feasible as a self-measurement device.

** sensitivity and specificity depend on the cut-off used, full details can be found in the HTA report [1], in general the sensitivity and specificity estimates relate to a cut-offs to detect early glaucoma.*

1. Burr JM et al. The clinical effectiveness and cost-effectiveness of screening for open angle glaucoma: a systematic review and economic evaluation. Health Technol Assess 2007;11:1-190.

2. Iwase et al. Performance of Frequency-Doubling Technology Perimetry in a Population-Based Prevalence Survey of Glaucoma. Ophthalmology 2007; 114:27–32.

3. Ya Xing Wang et al. Frequency-Doubling Threshold Perimetry in Predicting Glaucoma in a Population-Based Study Arch Ophthalmol. 2007;125(10):1402-1406

4. Mansberger et al. The results of Screening Frequency Doubling Perimetry Technology Perimetry in Different locations in the community. J Glaucoma 2007;1673-80

5. Iester M, Mikelberg FS, Drance SM. The effect of optic disc size on diagnostic precision with the Heidelberg retina tomograph. Ophthalmology 1997;104:545-8.

6. Medeiros FA, et al. Influence of disease severity and optic disc size on the diagnostic performance of imaging instruments in glaucoma. Invest Ophthalmol Vis Sci 2006;**110**:1145-50.

7. To´th et al. Accuracy of Combined GDx-VCC and Matrix FDT in a Glaucoma Screening Trial. J Glaucoma 2007;16:462–470
