# Supplementary material for: Developing the specifications of an Open Angle Glaucoma screening intervention in the United Kingdom: a Delphi approach
Source: BMC Health Serv Res. 2012 Dec 5;12:447. doi: 10.1186/1472-6963-12-447 (PMC3563574; doi:10.1186/1472-6963-12-447)
Supplement: Additional file 2 — Feedback to participants from the round one Delphi. A summary of the responses from round one sent to all participants with their round two questionnaires. [file 1472-6963-12-447-S2.pdf]

Developing the intervention for a future randomised controlled trial (RCT) of population screening for open angle glaucoma (OAG).
Feedback from Round 1

A multidisciplinary team was consulted on their views of screening interventions for OAG for the development of a future RCT. We had an excellent response rate, 31 questionnaires (82%) returned from 38 sent out from all the groups consulted.

A summary of the responses from sections A to C is provided. We are very grateful that many respondents took the time to write very detailed and helpful comments. These will not be reported here as these rich data will be explored and used to inform the next part of our research which will be semi-structured in depth interviews.

Also in round one, data were provided on combinations of tests, scenarios for screening strategies etc. These data will not be used in round 2, but will be used in the subsequent semi-structured interviews to determine the detail of the testing strategy, screening combinations, once the best bets of configurations of screening intervention are short listed by this Delphi approach.

SECTION A: Your views on feasible screening test strategies.

A1. TESTS OF VISUAL FUNCTION

93% (29/31) respondents thought that a test measuring visual function should be considered. However, 30 respondents rated the tests.

Summary of response for measures of functional loss (N = 30)

**Test**	**Rating scale – number of ratings (%)**				
	**0** (not suitable)	**1**	**2**	**3**	**4** (very suitable)
**FDT**		2 (7)	7 (23)	15 (50)	6 (20)
**OKP**	9 (30)	11 (37)	9 (30)	1 (3)	
**SAP**	3 (10)	2 (7)	10 (33)	11 (37)	4 (13)

Five respondents also mentioned Motion Displacement Test and one respondent suggested Blue/yellow perimetry (SWAP).

A1.1. Screening questionnaire asking for self report of risk factors

We would like to apologise for wrongly placing the screening questionnaire with tests of visual function. However, many respondents thought that this should be considered as a 'test' and some of the comments are detailed below.

Comments:

- 'Candidate screening questionnaire should include report of family history.' 'They are useful to identify the high risk population for screening'.
- 'Candidate screening questionnaire (self-report of risk factors) is not a test of visual function, but this may be an option as the first screening test, as identifying a target population based on a risk factor is not likely to be feasible'.
- 'Questionnaires may help to select most at risk population by identifying risk factors such as age family history and race. May be combined with other tests depending on score.'
- 'Questionnaire not likely to be useful alone but might be combined with any function tests.'

A2. Tests of structural loss

90% (28/31) respondents thought that a measure of optic disc /retinal nerve fibre layer loss be considered.

Summary of response for measures of structural loss (N = 28)

Test	Rating scale – number of ratings (%)				
	**0** (not suitable)	**1**	**2**	**3**	**4** (very suitable)
**Digital fundus photography**	1 (4)	5 (18)	6 (21)	10 (36)	6 (21)
**HRT**		4 (14)	11 (40)	7 (25)	6 (21)
**GDX-VCC**	1 (4)	9 (32)	8 (29)	6 (21)	4 (14)

Eight respondents suggested OCT as another test of structural loss and others were clinical bio microscopy of optic disk, gonioscopy in hyperopes, assessment of the disc with Volk lens and stereo photography.

A3. Measures of Intraocular Pressure (IOP).

90% (28/31) respondents thought that tonometry a measure of IOP should be considered.

.

Summary of response for measures of IOP (N = 28)

Test	Rating scale – number of ratings (%)				
	**0** (not suitable)	**1**	**2**	**3**	**4** (very suitable)
**GAT**	2 (7)	3 (11)	7 (25)	9 (32)	7 (25)
**I-Care**	5 (18)	3 (11)	10 (36)	6 (21)	4 (14)
**NCT**	4 (14)	4 (14)	4 (14)	12 (43)	4 (14)
**Tonopen**	4 (14)	6 (21)	10 (36)	4 (14)	4 (14)

One other suggestion was Reichert NCT or similar.

SECTION B: Your views on the target population.

B1. At what age would you start screening? (N = 31)

Age	Percentage
40	8 (26)
45	1 (3)
50	16 (52)
55	1 (3)
60	4 (13)
Other – 30 for black popn.	1 (3)

45% would consider screening on age alone, 39% would not and 16% were unsure.

B2. If you would screen on age and other risk factors, indicate all that would apply?

The numbers of respondents who would screen for the following risk factors is given.

Risk Factor	N
Black ethnic group	22
Diabetic	3
Myopic	9
Parent with OAG	24
Sibling OAG	23
Other	4

Other risk factors were –

- Any history of vasospasm such as migraine or Raynaud's phenomenon
- Previous high IOP, pseudoexfoliation
- Asians for angle – closure
- Child with OAG.

B3. Would you vary your choice of screening tests according to the population you were screening?

Only eight (26%) respondents would vary their choice of tests according to the population being screened.

SECTION C: **Screening site and operator**

C1. Which sites are suitable for the administration of a screening test? (N = 31)

Sites	Rating scale – number of ratings (%)				
	0 (not suitable)	1	2	3	4 (very suitable)
**Community optometric practice**	3 (10)	1 (3)	3 (10)	11 (36)	13 (42)
**General Practice**	2 (7)	5 (16)	11 (36)	7 (23)	6 (19)
**Home**	16 (52)	10 (32)	4 (13)	1 (3)	
**Hospital**	6 (19)	8 (26)	10 (32)	4 (13)	3 (10)
**Mobile van**	3 (10)	5 (16)	9 (29)	7 (23)	7 (23)
**Pharmacy**	5 (16)	12 (39)	7 (23)	3 (10)	4 (13)
**Supermarket**	7 (23)	11 (36)	4 (13)	6 (19)	3 (10)
**Workplace**	8 (26)	14 (45)	5 (16)	3 (10)	1 (3)

Other suggested sites were -

- Village health days' focused, free, local, can be locally advertised, local community can stimulate and control, especially if the local council pays (co-payment) for the service to the professional provider
- Shopping malls
- Pubs and gyms
- Polyclinic-meaning a health care complex with pharmacy etc serving several GP practice locations.
- NHS admin setting (need N3 NHS net connection not available currently unless GP practice or hospital NHS premises admin setting as clinical space at a premium) N3 connection will be with community optometry and pharmacy by end of 2010.
- Open access care centres for those not registered with GP
- Church/community hall
- Optic dispensary (if it were different from community optometric practice)
- Post office

C2. Who would provide the screening test? (N = 31)

Operator	Rating scale – number of ratings (%)				
	0 (not suitable)	1	2	3	4 (very suitable)
**Community optometrist**	3 (10)	2 (7)	1 (3)	12 (39)	12 (42)
**Nurse**	3 (10)	2 (7)	9 (29)	9 (29)	8 (26)
**Self testing**	19 (62)	9 (29)	2 (7)		1 (3)
**Specialist, glaucoma trained optometrist**	6 (19)	7 (23)	3 (10)	3 (10)	12 (39)
**Technician**	2 (7)	1 (3)	5 (16)	8 (26)	15 (48)

Other suggested providers were -

- Optimal: professional screening provider
- Physiotherapist, osteopath etc. sports coaches
- Resident in training
- Community optometrist plus technician

Based on these responses we would now like you to look at the next questionnaire and rate the combinations provided.
